# Disulfiram Sensitizes a Therapeutic-Resistant Glioblastoma to the TGF-β Receptor Inhibitor

**DOI:** 10.3390/ijms221910496

**Published:** 2021-09-28

**Authors:** Chan-Chuan Liu, Cheng-Lin Wu, Meng-Xuan Lin, Chun-I Sze, Po-Wu Gean

**Affiliations:** 1 Institute of Basic Medical Sciences, College of Medicine, National Cheng-Kung University, Tainan 701, Taiwan; sln4421@hotmail.com; 2 Institute of Clinical Medicine, College of Medicine, National Cheng Kung University Hospital, National Cheng-Kung University, Tainan 701, Taiwan; wujl.towalkwithwings@gmail.com; 3 Department of Pathology, College of Medicine, National Cheng Kung University Hospital, National Cheng-Kung University, Tainan 701, Taiwan; 4 Department of Cell Biology and Anatomy, College of Medicine, National Cheng-Kung University, Tainan 701, Taiwan; amber002091@hotmail.com.tw; 5 Department of Pharmacology, College of Medicine, National Cheng-Kung University, Tainan 701, Taiwan; 6 Department of Biotechnology and Bioindustry Sciences, National Cheng-Kung University, Tainan 701, Taiwan

**Keywords:** therapeutic resistance, ALDH activity, mesenchymal-like phenotype

## Abstract

Despite neurosurgery following radiation and chemotherapy, residual glioblastoma (GBM) cells develop therapeutic resistance (TR) leading to recurrence. The GBM heterogeneity confers TR. Therefore, an effective strategy must target cancer stem cells (CSCs) and other malignant cancer cells. TGF-β and mesenchymal transition are the indicators for poor prognoses. The activity of aldehyde dehydrogenases (ALDHs) is a functional CSC marker. However, the interplay between TGF-β and ALDHs remains unclear. We developed radiation-resistant and radiation-temozolomide-resistant GBM models to investigate the underlying mechanisms conferring TR. Galunisertib is a drug targeting TGF-β receptors. Disulfiram (DSF) is an anti-alcoholism drug which functions by inhibiting ALDHs. The anti-tumor effects of combining DSF and Galunisertib were evaluated by in vitro cell grow, wound healing, Transwell assays, and in vivo orthotopic GBM model. Mesenchymal-like phenotype was facilitated by TGF-β in TR GBM. Additionally, TR activated ALDHs. DSF inhibited TR-induced cell migration and tumor sphere formation. However, DSF did not affect the tumor growth in vivo. Spectacularly, DSF sensitized TR GBM to Galunisertib both in vitro and in vivo. ALDH activity positively correlated with TGF-β-induced mesenchymal properties in TR GBM. CSCs and mesenchymal-like GBM cells targeted together by combining DSF and Galunisertib may be a good therapeutic strategy for recurrent GBM patients.

## 1. Introduction

Glioblastoma (GBM) is the most malignant primary brain tumor in adults. The standard of care (SoC) of GBM consists of neurosurgical removal followed by radiotherapy, chemotherapy, radiochemotherapy, or a tumor treating field device, such as co-adjuvant therapy [1]. Temozolomide (TMZ) is the most common drug used in GBM chemotherapy. Radiation is a powerful approach to controlling tumor growth and significantly improves patient survival [2]. However, the benefit of radiochemotherapy varies in the case of GBM [3]. According to the World Health Organization classification of tumors in the central nervous system, in spite of receiving SoC, the median time to recurrence is about 7 months, and median survival is about 15 months [4]. Developing therapeutic resistance in residual GBM results in the failure of SoC and leads to poor prognoses [5]. GBM is derived from transformed astrocytes and other neural progenitor cells [6,7]. The heterogeneity of GBM confers therapeutic resistance.

Cancer stemness and epithelial–mesenchymal transition (EMT), or mesenchymal differentiation in GBM, play important roles in cancer progression [8,9,10,11]. TGF-β is a master molecule controlling a variety of cell physiologies, including EMT, tumor invasiveness, and metastasis [12]. The TGF-β signal transduces primarily via type I and type II serine/threonine kinase receptors (TRI and TRII). TGF-β-TRI/II complex phosphorylates Smad2 to activate the downstream cascade and regulate gene expression [13]. Upregulated and activated TGF-β signaling results in poor prognosis in GBM [14,15]. Blockade of TGF-β/Smad signaling sensitizes GBM to radiotherapy [16]. In addition, GBM patients with high levels of MD tend to have dismal outcomes and are more resistant to radiotherapy than those with low levels of MD [17].

The aldehyde dehydrogenase (ALDH) family regulates alcohol and fatty acid metabolism. Recently, upregulated or hyperactivated ALDHs have been shown to potentiate cancer stemness [18]. ALDH1 is defined as a newly recognized cancer stem cell marker. Evidence shows that ALDH regulates various malignant properties, such as cell migration, invasion, and drug resistance [19,20,21,22,23]. 

Although both TGF-β signaling and ALDHs are positive indicators for cancer malignancy, the crosstalk between ALDH and TGF-β remains a controversial topic. Furthermore, the interplay between TGF-β signaling and ALDH and its therapeutic potential remain unclear in terms of GBM resistance. Accordingly, we investigated the correlation of TGF-β signaling and ALDH as it relates to therapeutic resistance. We hypothesized that concurrent ALDH and TGF-β inhibition may be an effective strategy for suppressing therapeutic-resistant GBM both in vitro and in vivo.

## 2. Results

### 2.1. Developing Therapeutic Resistance in Glioblastoma Promotes TGF-β-Induced Mesenchymal-Like Phenotype

**Figure 1:**
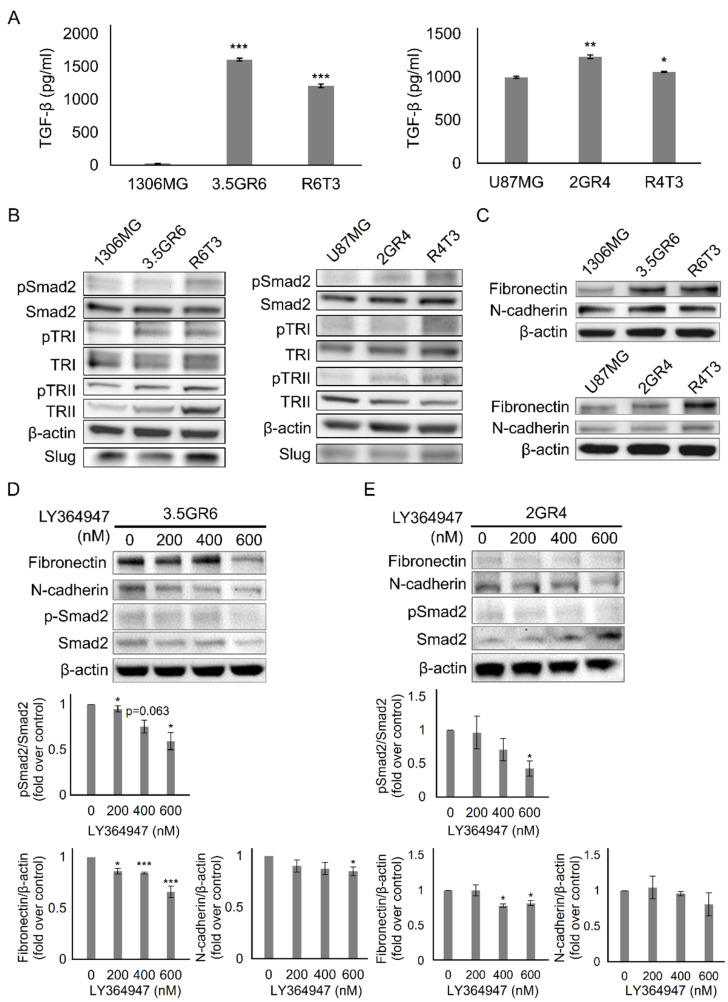
Developing therapeutic resistance promoted TGF-β-induced mesenchymal-like phenotype. (**A**) The concentration of TGF-β in the condition medium was measured using an enzyme-linked immunosorbent assay (ELISA). The histograms showing the secretion of TGF-β in 1306MG and U87MG; N = 4; (**B**) western blotting (WB) showing the expression of the downstream effectors of TGF-β, including phosphorylated and total TGF-β receptor I (TRI), phosphorylated and total TGF-β receptor II (TRII), phosphorylated and total Smad2, and Slug; (**C**) WB showing the expression of Fibronectin and N-cadherin; (**D**) TGF-β receptors of 1306MG were inhibited by LY364947 for 24 h. WB and histograms showing the ratio of pSmad2 and Smad2 and the protein expression of Fibronectin and N-cadherin; (**E**) TGF-β receptors of U87MG were inhibited by LY364947 for 24 h. WB and histograms showing the ratio of pSmad2 and Smad2 and the protein expression of Fibronectin and N-cadherin; the data were represented as mean ± standard error of the mean (SEM); * refers to a comparison with the control; *, *p* < 0.05; **, *p* < 0.01; ***, *p* < 0.001.

**Figure 2:**
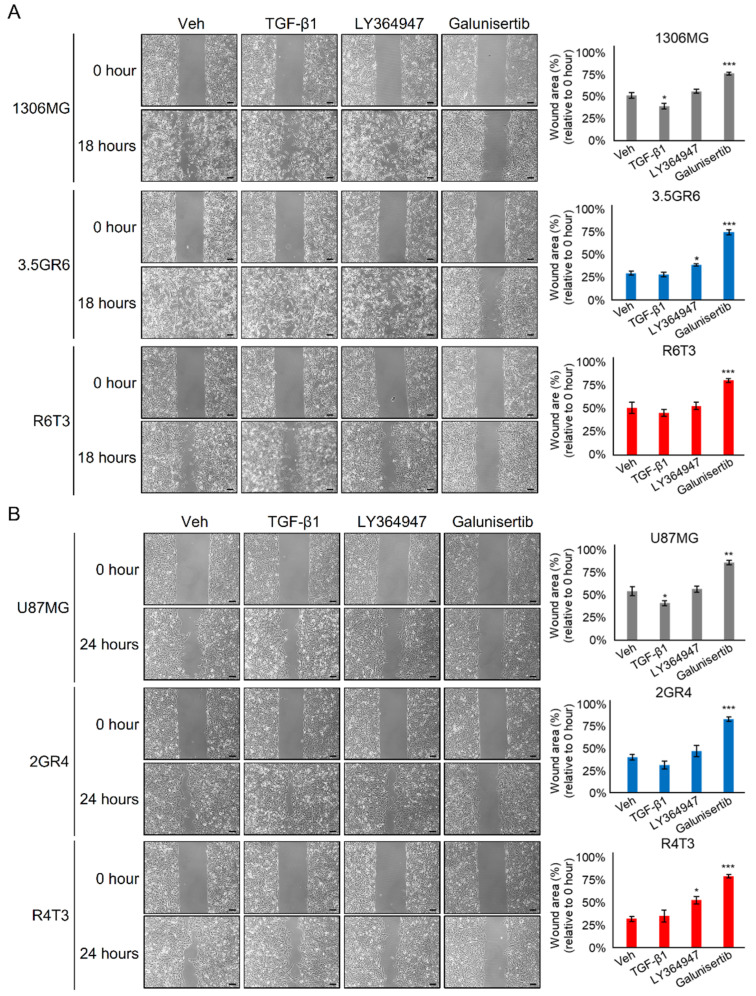
Suppressing TGF-β signaling reversed therapeutic resistance-facilitated cell motility. TGF-β signaling was blocked by both 4 μM LY364947 and 100 μM Galunisertib. Wound healing was used to evaluate cell migration. 0.1% DMSO was used as vehicle control (Veh). (**A**) Graphs and histograms showing the cell migration of 1306MG, radiation-resistant 3.5GR6, and radiation-temozolomide (TMZ)-resistant R6T3; N = 3 for 1306MG and 3.5GR6; N = 4 for R6T3; (**B**) graphs and the histograms showing the cell migration of U87MG, radiation-resistant 2GR4, and radiation-TMZ-resistant R4T3; N = 3 for U87MG; N = 4 for 2GR4 and R4T3; scale bar = 100 μm; mean ± SEM; * refers to a comparison with the control; *, *p* < 0.05; **, *p* < 0.01; ***, *p* < 0.001.

In our previous finding, the development of radiation resistance in glioblastoma (GBM) enhances TGF-β secretion and reduces temozolomide (TMZ) sensitivity [24]. Therefore, we sequentially challenged radiation-resistant (RR) GBM cells with TMZ to develop a radiation-TMZ-resistant (RTR) GBM cell model. RR GBM cells responding to TMZ dosages of 50, 100, 150, 200, and 250 μM were evaluated. A single treatment of 250 μM TMZ only inhibited the viability the RR GBM cells by about 30% (72.9 ± 2.46% for 1306MG R6T1; 62.4 ± 3.52% for U87MG R4T1). However, three consecutive treatments of 250 μM TMZ were unable to suppress RR GBM cell growth (85.5 ± 1.28% for 1306MG R6T3; 88.3 ± 2.15% for U87MG R4T3). Therefore, the cells with three TMZ treatments were defined as RTR GBM cells (1306MG R6T3 and U87MG R4T3) (Figure S1). TGF-β is a key regulator of the epithelial–mesenchymal transition (EMT) or mesenchymal differentiation. Both TGF-β and mesenchymal properties are malignant indicators in cancer progression. We hypothesized that TGF-β-induced mesenchymal transition would be enhanced in both RR and RTR GBM. We first examined the TGF-β secretions. In comparison with parental GBM cells, the secretion of TGF-β (25.7 ± 2.69 pg/mL vs. 1607.7 ± 19.16 pg/mL vs.1207.0 ± 27.33 pg/mL for 1306MG, 3.5GR6, and R6T3; 995.4 ± 10.98 pg/mL vs. 1234.1 ± 23.10 pg/mL vs. 1058.1 ± 8.56 pg/mL for U87MG, 2GR4, and R4T3) increased in both RR and RTR (Figure 1A). In addition, the downstream effectors of TGF-β, including TGF-β receptor I (TRI), TRII, Slug, and Snail were upregulated, and the phosphorylation on TRI, TRII, and Smad2 was enhanced in both RR and RTR (Figure 1B). RR and RTR increased the expression of the mesenchymal markers, N-cadherin and Fibronectin (Figure 1C). Furthermore, blockade of TGF-β signaling with 200, 400, and 600 nM of the TRs inhibitor LY364947 inhibited RR-induced phosphorylation on Smad2 and reduced the expression of N-cadherin and Fibronectin. In addition, we modulated the activity of TGF-β signaling through exogenous TGF-β1 or inhibiting TRs with LY364947 and Galunisertib. The results of MTT assay showed that LY364947 did not affect the cell viability of both parental and TR 1306MG and U87MG cells (Figure S2A). On the other hand, up to 500 μM Galunisertib was able to inhibit approximately 40% of cell viability (Figure S2B). Accordingly, we selected 4 μM LY364947 and 100 μM Galunisertib, which inhibited 20% of cell viability, for further investigation. As shown in Figure 2, activated TGF-β signaling by exogenous TGF-β1 only enhanced the cell motility of the parental GBM cells (51.8 ± 3.29% vs. 39.7 ± 3.26%, *p* = 0.0438 for 1306MG; 54.4 ± 5.18% vs. 41.0 ± 2.63%, *p* = 0.0424 for U87MG) while exogenous TGF-β1 did not affect the cell motility of therapeutic-resistant GBM cells (RR and RTR) (29.9 ± 2.17% vs. 28.8 ± 2.48%, *p* = 0.7663 for 3.5GR6, 50.5 ± 6.09% vs. 45.5 ± 3.68%, *p* = 0.3549 for R6T3; 40.7 ± 3.39% vs. 32.0 ± 4.27%, *p* = 0.0732 for 2GR4, 32.3 ± 2.91% vs. 35.4 ± 6.58%, *p* = 0.6075 for R4T3). On the other hand, blockade of TRs by LY364947 or Galunisertib suppressed cell migration in both the parental and therapeutic-resistant GBM cells. Thus, the development of therapeutic resistance facilitated TGF-β-induced mesenchymal properties.

### 2.2. The Role of ALDH in Therapeutic Resistance

**Figure 3:**
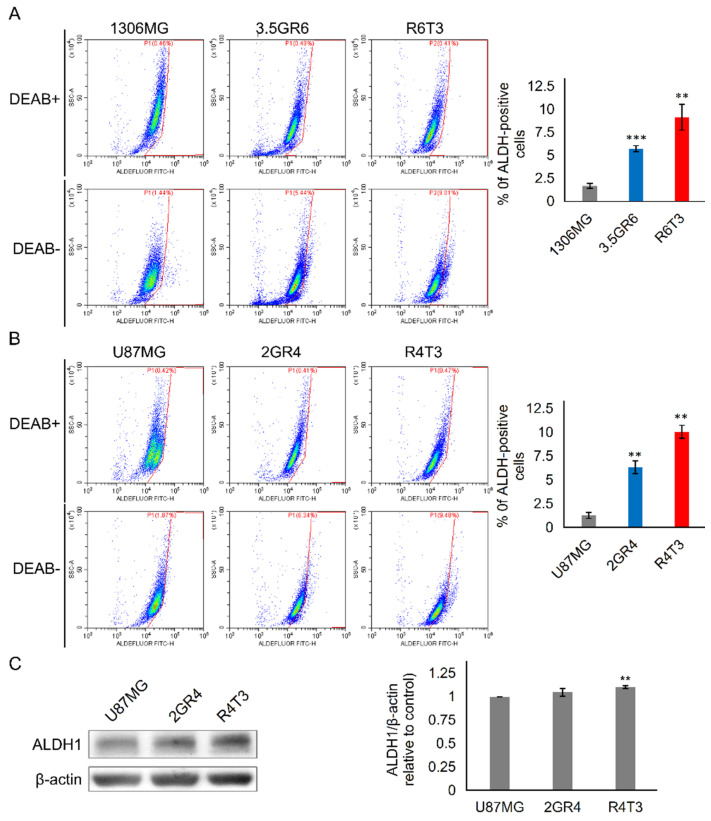
Developing therapeutic resistance-activated aldehyde dehygrogenases. The activity of aldehyde dehygrogenases (ALDHs) was measured using the ALDEFLUOR assay with flow cytometry. N, N-diethylaminobenzaldehyde (DEAB) was used to inhibit ALDH. (**A**) Representative flow cytometry plots and histograms showing the activity of ALDHs in 1306MG, radiation-resistant 3.5GR6, and radiation-TMZ-resistant R6T3; N = 3 for 3.5GR6; N = 4 for 1306MG; N = 5 for R6T3; (**B**) representative flow cytometry plots and histograms showing the activity of ALDHs in U87MG, radiation-resistant 2GR4, and radiation-TMZ-resistant R4T3; N = 3; (**C**) WB and histogram showing the protein expression of ALDH1A1 of U87MG, radiation-resistant 2GR4, and radiation-TMZ-resistant R4T3; N = 3; mean ± SEM; * indicates comparison with parental 1306MG or U87MG; **, *p* < 0.01; ***, *p* < 0.001.

Cancer stem cell properties play a role in cancer progression. The activity and expression of ALDHs are the indicators of cancer stemness. ALDH activity was measured using an ALDEFLUOR assay. We found that developing RR and RTR activated ALDHs (Figure 3A,B). Also, the expression of ALDH1 was upregulated in the RR and RTR GBM cells (1 ± 0 vs. 1.05 ± 0.04 and 1.10 ± 0.34, *p* = 0.0153 and 0.0017 for U87MG vs. 2GR4 and R4T3) (Figure 3C). Disulfiram (DSF), the pan-ALDHs inhibitor, is an old drug used for alcoholism. DSF as a single or co-adjuvant treatment for cancers increases anti-tumor efficacy [25,26]. Additionally, DSF is able to enhance radiation responses and chemotherapy sensitivity in cancers, but its targets and the underlying mechanism are not known [27,28]. Therefore, we evaluated the cell response to different doses of DSF (Figure S3A). A 20% dose of growth inhibition (GI_20_) was selected for further investigation (8 μM for 1306MG, 3.5GR6, and R6T3; 150 nM for U87MG, 2GR4, and R4T3). NCT-501, a specific inhibitor of ALDH1A1, was selected to elucidate the role of ALDH1 in therapeutic resistance. We first evaluated the effects of NCT-501 on cell growth. As shown in Figure S3B, NCT-501 did not affect the cell viability.

**Figure 4:**
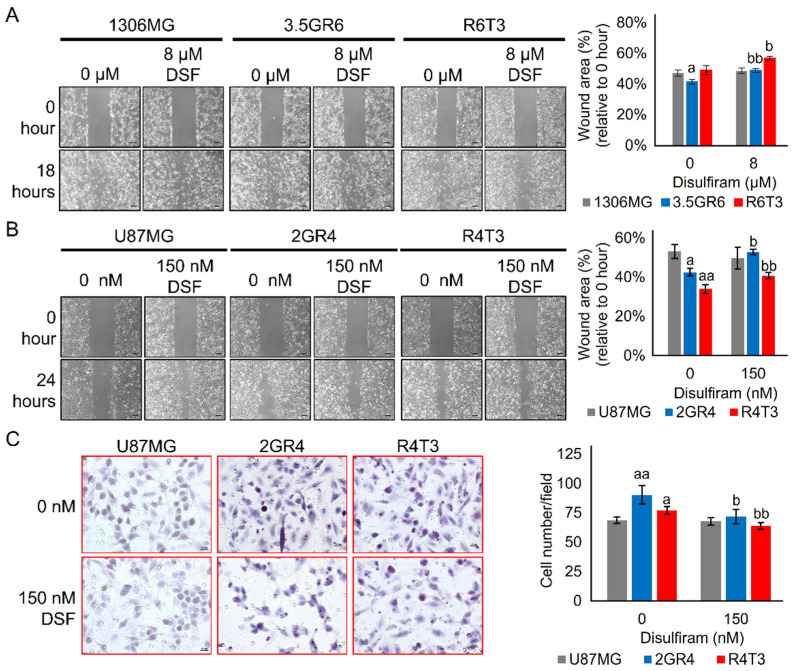
Disulfiram inhibited therapeutic resistance-induced cell mobility. A 20% dose of Disulfiram (DSF) growth inhibitor was selected. 0.1% DMSO was used as solvent control (labelled as 0 μM). The cell mobility was evaluated using a two-dimensional (2D) wound healing assay and a three-dimensional (3D) Transwell assay. (**A**) Graphs of the wound and histogram showing the inhibitory effects of DSF on 2D cell mobility of 1306MG, radiation-resistant 3.5GR6, and radiation-TMZ-resistant R6T3; N = 3; scale bar = 100 μm; (**B**) graphs and histogram showing the inhibitory effect of DSF on 2D cell mobility of U87MG, radiation-resistant 2GR4, and radiation-TMZ-resistant R4T3; N = 3; scale bar = 100 μm; (**C**) graphs showing the migratory cells and histogram indicating the cell mobility of the inhibitory effect of DSF on 3D cell mobility of U87MG, radiation-resistant 2GR4, and radiation-TMZ-resistant R4T3; N = 5; scale bar = 20 μm; mean ± SEM; a refers to a comparison with parental 1306MG or U87MG; b refers to a comparison with the control; a and b, *p* < 0.05; aa and bb, *p* < 0.01.

ALDHs may enhance migration and invasion abilities and in turn contribute to EMT [19,20,21]. Therefore, cell mobility was evaluated using a two-dimensional (2D) wound healing assay and a three-dimensional (3D) Transwell assay. Interestingly, the DSF dose of GI_20_ did not affect the 2D cell migration of parental 1306MG and U87MG (47.3 ± 2.1% vs. 48.7 ± 1.7%, *p* = 0.5908 for 1306MG; 51.5 ± 3.8% vs. 49.7 ± 5.5%, *p* = 0.7927 for U87MG), while it significantly inhibited therapeutic resistance-induced cell motility (41.4 ± 1.6% vs. 48.9 ± 1.2%, *p* = 0.0019 for 3.5GR6, 49.2 ± 3.0% vs. 56.9 ± 1.3%, *p* = 0.0361 for R6T3; 43.5 ± 2.1% vs. 52.9 ± 1.3%, *p* = 0.0016 for 2GR4). However, DSF did not affect the 2D mobility of RTR U87MG R4T3 (33.9 ± 2.2% vs. 38.4 ± 2.0%, *p* = 0.1496) (Figure 4A,B). In the 3D migration results, the DSF dose of GI_20_ did not affect the 3D motility of the parental U87MG (68.9 ± 2.8 vs. 67.85 ± 3.2, *p* = 0.950), but it reduced the 3D migration ability of both RR and RTR U87MG (90.4 ± 7.8 vs. 71.85 ± 6.1, *p* = 0.0117 for 2GR4, 77.2 ± 3.4 vs. 63.85 ± 2.8, *p* = 0.0001 for R4T3) (Figure 4C).

On the other hand, we also examined whether specifically inhibiting NCT-501 attenuated cell motility. NCT-501 did not affect the cell migration of parental 1306MG. In contrast, 300 nM NCT-501 was able to inhibit the cell motility of RR 1306MG 3.5GR6 (32 ± 3.9% vs. 44 ± 3.9%, *p* = 0.0108) and RTR 1306MG R6T3 (46 ± 2.6% vs. 59 ± 3.1%, *p* = 0.0216) (Figure S4A). 100 nM NCT-501 was able to reduce the 2D migration ability of parental U87MG (43 ± 2.36% vs. 52 ± 1.60%, *p* = 0.0037), RR 2GR4 (34 ± 1.40% vs. 47 ± 2.80%, *p* = 0.0005), and RTR R4T3 (47 ± 0.63% vs. 53 ± 2.13%, *p* = 0.0597) (Figure S4B). Therefore, 300 nM NCT-501 for 1306MG and 100 nM NCT-501 U87MG were selected for further investigation. Otherwise, the inhibitory efficacy of DSF and NCT-501 on ALDHs was measured using the ALDEFLUOR assay with flow cytometry. The DSF dose of GI_20_ and the selected dose of NCT-501 inhibited about 20% of the ALDH activity (Figure S5).

### 2.3. Combining TGF-β Signaling Inhibition and ALDH Inactivation Inhibits Cell Migration and the Growth of Tumor Spheres

**Figure 5:**
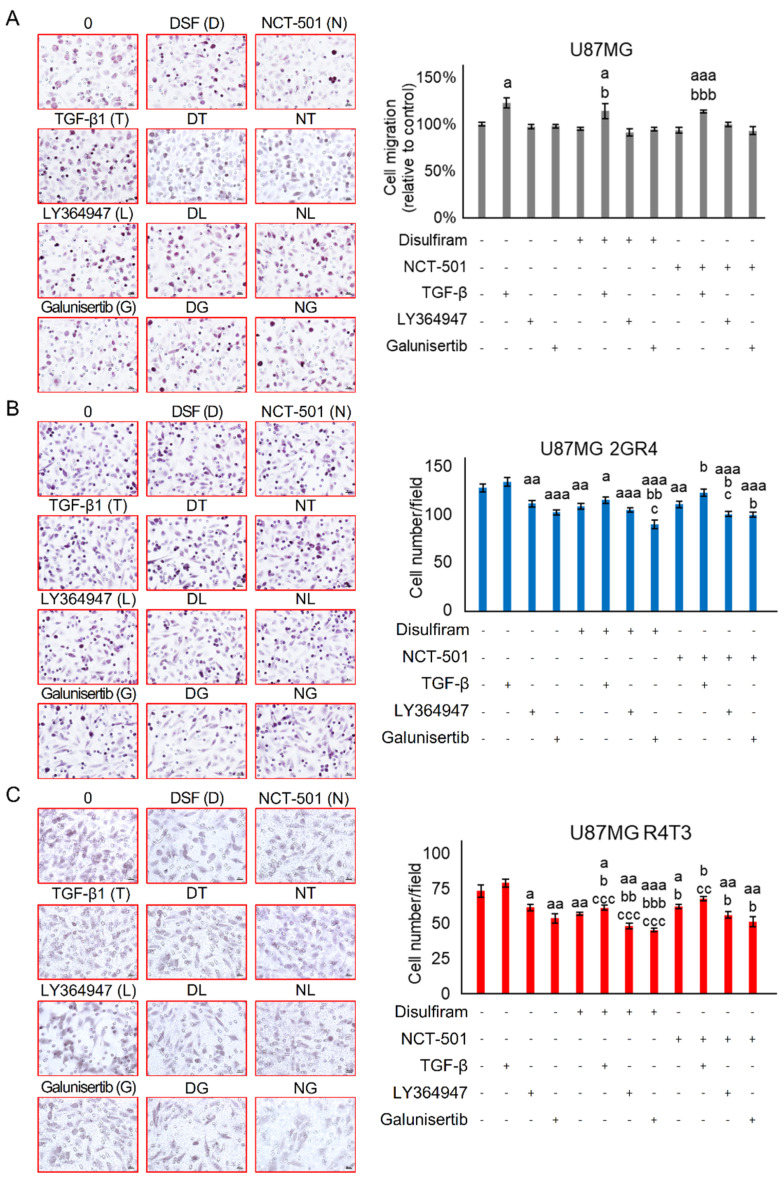
Combining ALDH and TGF-β inhibition suppresses the cell mobility. The cell mobility was evaluated using a 3D Transwell assay. 150 nM DSF, 100 nM NCT-501, 10 ng/mL TGF-β, 4 μM LY364947, and 100 μM Galunisertib were selected to treat the cells for 9 h. The “+” means with the treatment while the “-” means without the treatment. (**A**) Graphs showing the migratory cells and histogram indicating the cell mobility of U87MG under the various treatments; N = 6; (**B**) graphs showing the migratory cells and histogram indicating the cell mobility of radiation-resistant U87MG 2GR4 under the various treatments; N = 6; (**C**) graphs showing the migratory cells and histogram indicating the cell mobility of radiation-TMZ-resistant U87MG R4T3 under the various treatments; N = 6; scale bar = 20 μm; mean ± SEM; a refers to a comparison with the control; b refers to a comparison with sole DSF or NCT-501; c refers to a comparison with sole TGF-β1 or LY364947 or Galunisertib; a, b, c, *p* < 0.05; aa, bb, cc, *p* < 0.01; aaa, bbb, ccc, *p* < 0.001.

Due to high correlations among ALDH activity, TGF-β-induced MD, and therapeutic resistance, we hypothesized that combining the inhibition of ALDH and TGF-β signaling could lead to better therapeutic effects than single treatments with any of them alone. First, we examined the inhibitory effect of combined treatment on cell mobility using a Transwell migration assay. Exogenous TGF-β only increased the migratory cells in parental U87MG cells (105.0 ± 2.1 vs. 129.2 ± 5.7, *p* = 0.0017) (Figure 5A); however, it did not affect the cell mobility of RR U87MG 2GR4 (127.4 ± 4.2 vs. 133.6 ± 4.2, *p* = 0.3045) (Figure 5B) and RTR U87MG R4T3 (73.3 ± 4.4 vs. 79.1 ± 2.9, *p* = 0.2865) (Figure 5C). Meanwhile, the blockade of TRs by LY364947 or Galunisertib inhibited resistance-induced mobility in RR U87MG 2GR4 (127.4 ± 4.2 vs. 111.1 ± 3.4, *p* = 0.0070 for LY364947; vs. 102.2 ± 2.7, *p* < 0.001 for Galunisertib) (Figure 5B) and RTR U87MG R4T3 (73.3 ± 4.4 vs. 61.5 ± 2.3, *p* = 0.0334 for LY364047; vs. 53.8 ± 3.2, *p* = 0.0022 for Galunisertib) (Figure 5C). Combining DSF and Galunisertib led to better inhibitory effects on cell mobility than the use of either ALDH or TGF-β signaling inhibition alone in RR U87MG 2GR4 (89.3 ± 4.4 vs. 108.2 ± 2.7, *p* = 0.0019 for DSF, *p* = 0.0224 for Galunisertib) (Figure 5B) and RTR U87MG R4T3 (57.2 ± 0.9 vs. 45.5 ± 1.2, *p* < 0.001 for DSF, *p* = 0.0311) (Figure 5C). Exogenous TGF-β was able to rescue the inhibitory effect of NCT-501 on the cell motility of RR U87MG 2GR4 (110.3 ± 3.4 vs. 122.5 ± 4.1, *p* = 0.0329) (Figure 5B) and RTR U87MG R4T3 (62.4 ± 1.6 vs. 67.9 ± 1.8, *p* = 0.0322) (Figure 5C); however, it did not alter the effects of DSF on the cell migration of RR U87MG 2GR4 (108.2 ± 2.7 vs. 114.6 ± 3.4, *p* = 0.1534) (Figure 5B) and RTR U87MG R4T3 (57.2 ± 0.9 vs. 60.7 ± 1.7, *p* = 0.1006) (Figure 5C).

**Figure 6:**
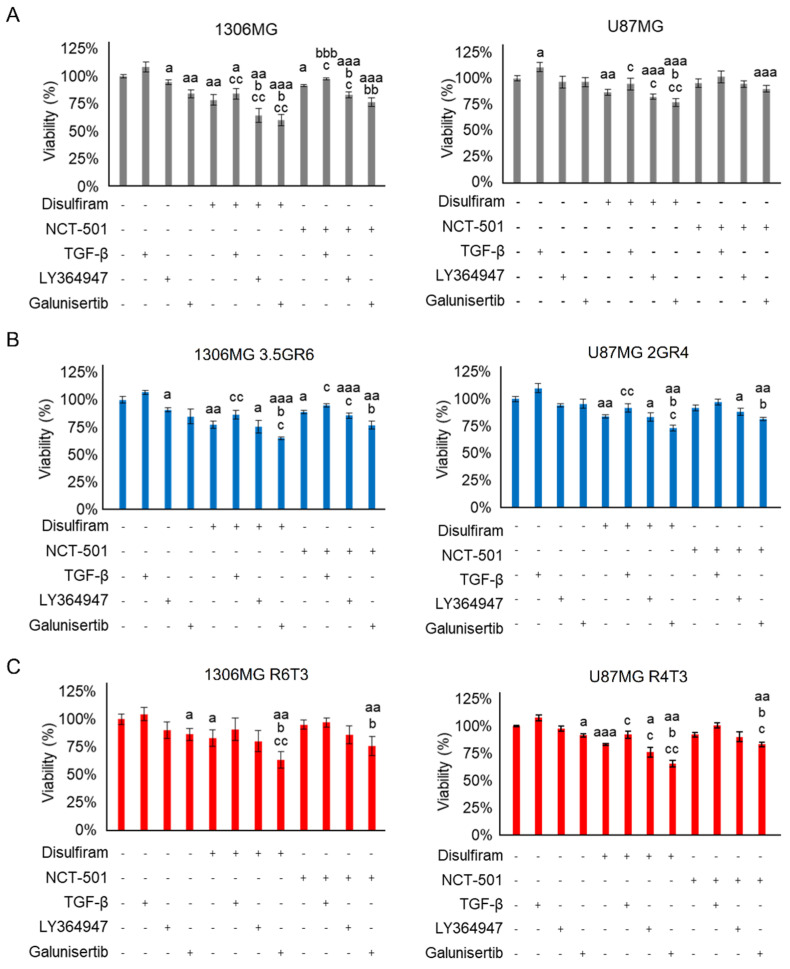
Combining ALDH and TGF-β inhibition represses cell growth. A Trypan blue exclusion assay was used to evaluate the cell proliferation. 150 nM DSF and 100 nM NCT-501 for U87MG, 8 μM DSF and 300 nM NCT-501 for 1306MG, 10 ng/mL TGF-β, 4 μM LY364947, and 100 μM Galunisertib were used to treat cells for 24 h. The “+” means with the treatment while the “-” means without the treatment. (**A**) Histograms showing the cell viability of parental 1306MG and U87MG; N = 4; (**B**) histograms showing the cell viability of radiation-resistant 1306MG 3.5GR6 and U87MG 2GR4; N = 5 for 3.5GR6; N = 3 for 2GR4; (**C**) histograms showing the cell viability of radiation-TMZ-resistant 1306MG R6T3 and U87MG R4T3; N = 3; mean ± SEM; a refers to a comparison with the 0 control; b refers to a comparison with sole DSF or NCT-501; c refers to a comparison with sole TGF-β or LY364947 or Galunisertib; a, b, c, *p* < 0.05; aa, bb, cc, *p* < 0.01; aaa, bbb, *p* < 0.001.

Next, we evaluated the cytotoxic effect of sole DSF, NCT-501, LY364947, Galunisertib and combined treatment on parental, RR, and RTR GBM cells. In parental GBM cells, exogenous TGF-β1 only promoted U87MG growth (100.0 ± 2.5% vs. 110.7 ± 4.5%, *p* = 0.0354) (Figure 6A). TGF-β1 did not affect cell growth of RR and RTR 1306MG and U87MG (Figure 6B,C). Inhibiting TGF-β signaling by LY364947 or Galunisertib was able to inhibit 10% of cell growth. Combining DSF and Galunisertib showed higher cytotoxicity to GBM than sole treatment, while exogenous TGF-β was not able to reverse the growth inhibition of DSF. DSF desensitized GBM cells to TGF-β1 in parental 1306MG (108.5 ± 4.5% vs. 84.3 ± 5.0%, *p* = 0.0016) and U87MG (110.7 ± 4.5% vs. 94.4 ± 5.7%, *p* = 0.0209) (Figure 6A), RR 1306MG 3.5GR6 (107.0 ± 1.9% vs. 86.5 ± 4.0%, *p* = 0.0046) and U87MG 2GR4 (110.1 ± 4.0% vs. 92.0 ± 3.8%, *p* = 0.0314) (Figure 6B), and RTR U87MG R4T3 (107.7 ± 2.8% vs. 92.3 ± 3.2%, *p* = 0.0231) (Figure 6C). Also, combining NCT-501 with either LY364947 or Galunisertib led to cytotoxicity. In addition, according to the combination index, combining DSF and Galunisertib showed slightly synergistic effects on RR 1306MG 3.5GR6 (CI = 0.895) and RTR 1306MG R6T3 (CI = 0.897), and it appeared to have synergistic effects on RR U87MG cells (CI = 0.672). However, combining DSF and Galunisertib had nearly additive effects on RTR U87MG R4T3 cells (CI = 0.970). Conversely, combining DSF and Galunisertib showed moderate antagonistic effects on parental 1306MG (CI = 1.203) and U87MG cells (CI = 1.333) (Table 1).

**Figure 7:**
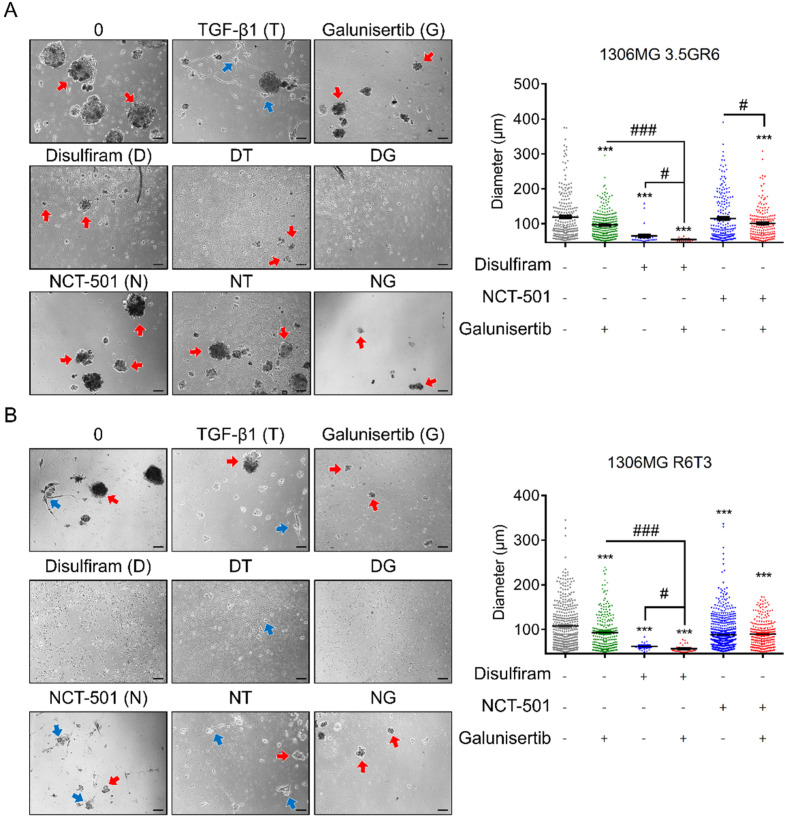
Combining ALDH inhibition and Galunisertib restrains the growth of tumor spheres. The 3D tumor sphere formation assay was used to evaluate the inhibitory effects of combining ALDH inhibition and Galunisertib on the growth of tumor spheres. The lengths of the tumor spheres were calculated. The “+” means with the treatment while the “-” means without the treatment. (**A**) Graphs and histogram showing the ability of radiation-resistant 1306MG 3.5GR6 to form tumor spheres; (**B**) graphs and histogram showing indicating the ability of radiation-TMZ-resistant 1306MG R6T3 to form tumor spheres; the red arrows indicate the tumor spheres; the blue arrows indicate the cells with the growth of processes; scale bar = 100 μm; mean ± SEM; * refers to a comparison with the control; # refers to a comparison with sole Galunisertib or DSF or NCT-501; * and #, *p* < 0.05; *** and ###, *p* < 0.001.

Also, we evaluated whether concurrent inhibition of ALDH and TGF-β receptors was able to inhibit the growth of tumor spheres. RR 1306MG 3.5GR6 and RTR 1306MG R6T3 were able to the growth of tumor spheres under 3D conditions. Furthermore, RTR 1306MG R6T3 cells not only formed spheres but grew processes under 3D conditions. Exogenous TGF-β1 promoted the processes formation, and Galunisertib reduced the growth of tumor spheres in RR 1306MG 3.5GR6 (119.0 ± 5.1 μm vs. 96.6 ± 2.5 μm, *p* < 0.001) (Figure 7A) and RTR 1306MG R6T3 (107.8 ± 0.6 μm vs. 93.3 ± 2.3 μm, *p* < 0.001) (Figure 7B). Inhibiting ALDHs with DSF led to suppression of the formation and growth of RR 1306MG 3.5GR6 tumor spheres (vs. 65.3 ± 5.6 μm, *p* < 0.001) (Figure 7A) and RTR 1306MG R6T3 (vs. 62.2 ± 1.9 μm *p* < 0.001) (Figure 7B). However, specifically inhibiting ALDH1A1 with NCT-501 only reduced the diameter of the RTR 1306MG R6T3 tumor spheres (vs. 88.2 ± 0.1 μm, *p* < 0.001) (Figure 7B). Combining DSF with Galunisertib led to better inhibitory effects in terms of tumor sphere than DSF and Galunisertib in RR 1306MG 3.5GR6 alone (vs. 54.8 ± 0.9 μm, *p* = 0.0425 for DSF, and *p* < 0.001 for Galunisertib) (Figure 7A) and RTR 1306MG R6T3 (vs. 57.1 ± 0.3 μm, *p* = 0.0354 for DSF, and *p* < 0.001 for Galunisertib) (Figure 7B). However, combining NCT-501 and Galunisertib led to better inhibitory effects on tumor sphere growth than NCT-501 alone in RR 1306MG 3.5GR6 (vs. 100.7 ± 3.7 μm, *p* = 0.0122) (Figure 7A). Accordingly, combining DSF and Galunisertib led to better inhibitory effects than the use of DSF or Galunisertib alone.

### 2.4. Disulfiram Sensitizes Resistant GBM to Galunisertib

**Figure 8:**
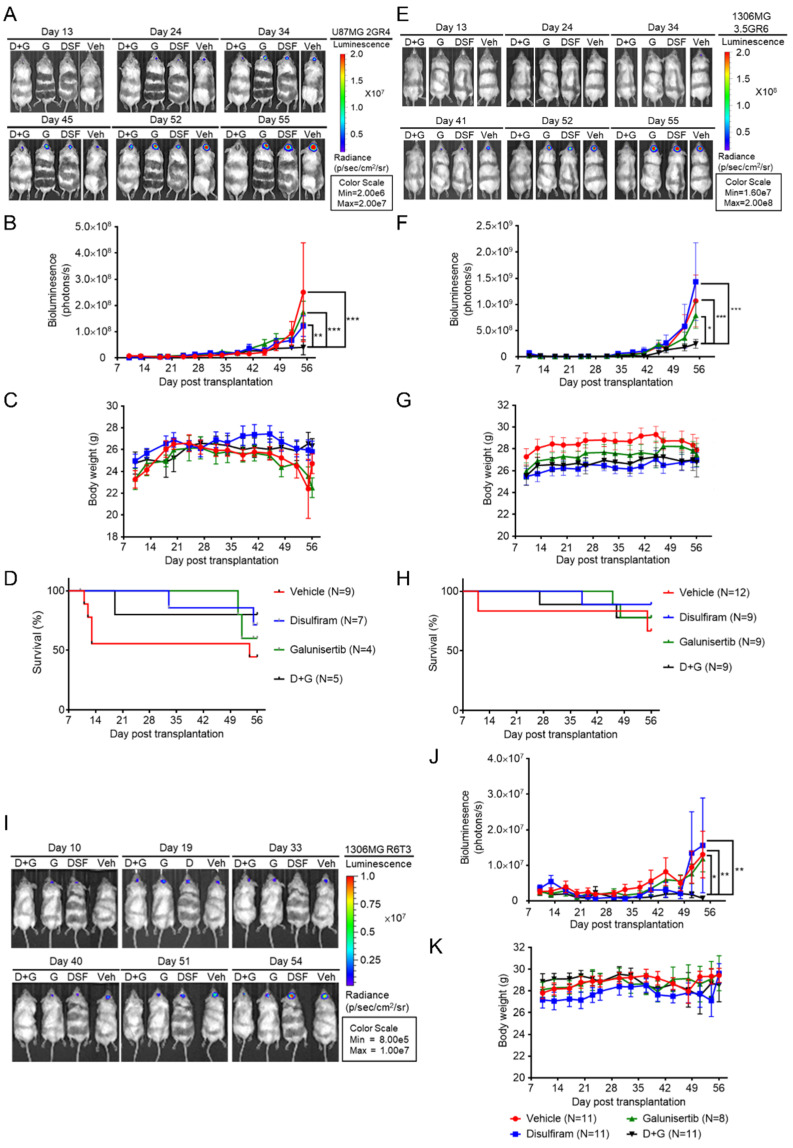
Combining DSF and Galunisertib shows better anti-tumor efficacy than sole treatments. The Luciferase (Luc)-expressing radiation-resistant U87MG 2GR4, or radiation-resistant 1306MG 3.5GR6, or radiation-TMZ-resistant 1306MG R6T3 cells (5 × 105 cells in 2 μL PBS) were injected into the striatum of the brain of NOD-SCID mice. At day 10 post-transplantation, DSF (50 mg/kg), Galunisertib (75 mg/kg), DSF (50 mg/kg) + Galunisertib (75 mg/kg), or DMSO as a vehicle was injected intraperitoneally twice weekly into the mice. Bioluminescence was used to monitor tumor growth with the IVIS-200 imaging system twice weekly and tumor growth was observed for 45 days. (**A**) Graphs showing the in vivo tumor growth of radiation-resistant U87MG 2GR4 cells; (**B**) histogram indicating the bioluminescent photons of tumor cells; (**C**) histogram showing the changes in body weight; (**D**) Kaplan–Meier survival curve of the vehicle-, DSF-, G-, and D + G-treated mice; (**E**) graphs showing the in vivo tumor growth of radiation-resistant 1306MG 3.5GR6 cells; (**F**) histogram indicating the bioluminescent photons of tumor cells; (**G**) histogram showing the changes in body weight; (**H**) Kaplan–Meier survival curve of the vehicle-, DSF-, G-, and D + G-treated mice; (**I**) graphs showing the in vivo tumor growth of radiation-TMZ-resistant 1306MG R6T3 cells; (**J**) histogram indicating the bioluminescent photons of tumor cells; (**K**) histogram showing the changes in body weight; mean ± SEM; * refers to a comparison with the D + G treatment; *, *p* < 0.05; **, *p* < 0.01; ***, *p* < 0.001.

In order to examine the anti-tumor effects and toxicity of DSF and Galunisertib (G) alone, and that when combining DSF and Galunisertib (D + G), we developed orthotopic xenograft GBM model by injecting luciferase-expressing therapeutic-resistant GBM cells into the striatum of NOD/SCID mice. At day 10 post-transplantation, the mice were treated twice weekly with DSF, G, D + G, or DMSO as a vehicle (Veh). As shown in Figure 8A, in comparison with Veh, DSF and G did not affect the in vivo growth of RR U87MG 2GR4 cells, while D + G significantly suppressed the in vivo growth of RR U87MG 2GR4 cells (Figure 8A). A two-way ANOVA revealed a main effect of DSF, G, D + G vs. Veh (F (3, 150) = 3.571, *p* = 0.0156), interaction (F (39, 150) = 1.260, *p* = 0.1647), and days after treatment (F (13, 150) = 11.79, *p* < 0.0001) (Figure 8B). Also, comparing Veh-treated groups, DSF, G, or D + G did not affect the body weight of the mice. A two-way ANOVA revealed a main effect of DSF, G, D + G vs. Veh (F (3, 157) = 4.851, *p* = 0.0030), interaction (F (42, 157) = 0.4747, *p* = 0.9971), and days after treatment (F (14, 157) = 2.766, *p* = 0.0011) (Figure 8C). In addition, a Kaplan–Meier analysis of the survival of the Veh-, DSF-, G-, and D + G-treated mice is presented in Figure 8D. The median survival of Veh-treated mice was 54 days while the median survival of DSF-, G-, and D + G-treated mice were not defined. In order to exclude the cell line-specific and resistance-specific effects, the luciferase-expressing RR 1306MG 3.5GR6 and RTR 1306MG R6T3 cells were injected intracranially into the NOD/SCID mice. The same procedures of IVIS observation and the treatments of Veh, DSF, G, and D + G were applied to the RR 1306MG 3.5GR6 and RTR 1306MG R6T3 cells in vivo growth. As shown in Figure 8E, compared with the Veh-treated groups, DSF and G did not affect tumor growth while D + G significantly delayed the in vivo tumor growth of RR 1306MG 3.5GR6. Furthermore, D + G showed better anti-tumor effects on RR 1306MG 3.5GR6 in vivo than DSF or Galunisertib alone (Figure 8E). A two-way ANOVA revealed a main effect of DSF, G, D + G vs. Veh (F (3, 443) = 2.843, *p* = 0.0375), interaction (F (39, 443) = 0.8691, *p* = 0.6968), and days after treatment (F (13, 443) = 11.89, *p* < 0.0001) (Figure 8F). In comparison with the Veh-treated groups, DSF, G, or D + G did not affect the body weight of the mice (Figure 8G). However, the survival curve of DSF, G, D + G, and Veh did not show the differences (Figure 8H). The similar phenomena were found in Figure 8I, only D + G was able to inhibit the in vivo growth of RTR 1306MG R6T3 cells while Veh, DSF, G did not affect the tumor growth. A two-way ANOVA revealed a main effect of DSF, G, D + G vs. Veh (F (3, 418) = 5.795, *p* < 0.0001), interaction (F (39, 418) = 0.7455, *p* = 0.8694), and days after treatment (F (13, 418) = 4.255, *p* < 0.0001) (Figure 8J). Also, DSF, G, or D + G did not affect the body weight of the mice (Figure 8K).

### 2.5. The Interplay between TGF-β Signaling and ALDH

**Figure 9:**
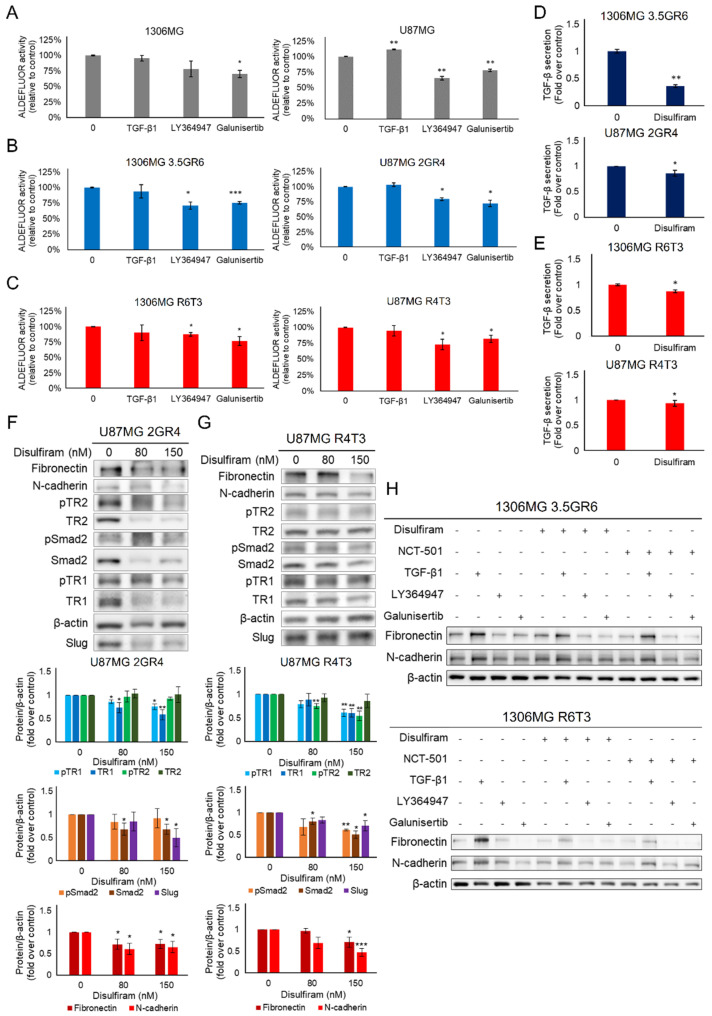
Blockade of ALDH by DSF suppresses TGF-β-regulated mesenchymal-like phenotype. An ALDEFLUOR assay was performed to evaluate the effects of modulating TGF-β signaling on ALDH activity. 10 ng/mL TGF-β1, 4 μM LY364947, and 100 μM Galunisertib were used to manipulate the TGF-β signaling activity. The “+” means with the treatment while the “-” means without the treatment. (**A**) Histograms showing the activity of ALDHs in parental 1306MG and U87MG; N = 4 for 1306MG; N = 3 for U87MG; (**B**) Histograms showing the activity of ALDHs in radiation-resistant 1306MG 3.5GR6 and U87MG 2GR4; N = 4 for 3.5GR6 and 2GR4; (**C**) Histograms showing the activity of ALDHs in radiation-TMZ-resistant 1306MG R6T3 and U87MG R4T3; N = 4 for R6T3; N = 5 for R4T3; (**D**) ELISA was used to measure the TGF-β secretions. 8 μM and 150 nM DSF were used to inhibit ALDHs in 1306MG and U87MG, respectively. The histograms showing TGF-β secretions in radiation-resistant 1306MG 3.5GR6 and U87MG 2GR4; N = 3 for 3.5GR6; N = 4 for 2GR4; (**E**) histograms showing the TGF-β secretions in radiation-TMZ-resistant 1306MG R6T3 and U87MG R4T3; N = 3 for R6T3; N = 4 for R4T3; (**F**) the cells were subjected to DSF for 24 h. The protein expression of the downstream effectors of TGF-β and mesenchymal markers was measured using WB. The WB and histograms showing the protein expression of phosphorylated and total TRI, TRII, Smad2, the transcription factor Slug, N-cadherin, and Fibronectin in radiation-resistant U87MG 2GR4; N = 3; (**G**) the WB and histograms showing the expression of phosphorylated and total TRI, TRII, Smad2, the transcription factor Slug, N-cadherin, and Fibronectin in radiation-TMZ-resistant U87MG R4T3; N = 3; (**H**) the WB showing the expression of N-cadherin and Fibronectin under treatment for 24 h in radiation-resistant 1306MG 3.5GR6 and radiation-TMZ-resistant 1306MG R6T3; mean ± SEM; * refers to a comparison with the control; *, *p* < 0.05; **, *p* < 0.01; ***, *p* < 0.001.

In order to clarify the crosstalk between TGF-β signaling and ALDHs, we first examined whether TGF-β signaling regulated ALDHs. Activating TGF-β signaling by exogenous TGF-β1 only enhanced the activity of ALDHs in parental U87MG cells (100 ± 0.32% vs. 112 ± 0.65%, *p* > 0.001), but it did not affect the ALDHs in parental 1306MG cells (100 ± 0.65% vs. 96 ± 4.55%, *p* = 0.386) (Figure 9A). In RR and RTR GBM, exogenous TGF-β1 did not alter the activity of ALDHs (100 ± 0.46% vs. 94 ± 10.90%, *p* = 0.6120 for 3.5GR6; 100 ± 0.62% vs. 103 ± 2.78%, *p* = 0.3532 for 2GR4; 100 ± 0.32% vs. 90 ± 12.53%, *p* = 0.4720 for R6T3; 100 ± 0.43% vs. 95 ± 8.06%, *p* = 0.5094 for R4T3) (Figure 9B,C). Therefore, TGF-β may not be the upstream regulator of ALDHs. On the other hand, inhibition of TGF-β receptors by either LY364947 or Galunisertib significantly reduced the activity of ALDHs in parental, RR, and RTR GBM cells (Figure 9A–C).

Conversely, we evaluated whether ALDH inhibition by DSF was able to inhibit therapeutic resistance-induced TGF-β signaling. As shown in Figure 9D,E, DSF suppressed the secretion of TGF-β in the RR and RTR GBM cells. DSF primarily reduced the phosphorylation of TRI. Also, it downregulated the expression of TRI in the RR and RTR GBM cells. In the RTR U87MG cells, DSF also inhibited the phosphorylation of TRII. In terms of intracellular signal transduction, DSF not only inhibited the phosphorylation of Smad2 in the RTR U87MG cells, but directly reduced the expression of Smad2 in the RR and RTR U87MG cells. In addition, the dose of DSF GI_20_ downregulated the transcription factor Slug and reversed therapeutic resistance-induced MD by decreasing N-cadherin and Fibronectin (Figure 9F,G). Furthermore, DSF and NCT-501 were able to de-sensitize RR and RTR GBM cells to exogenous TGF-β. Combining the inhibitory effects of the ALDHs and TGF-β receptors efficiently decreased the expression of mesenchymal N-cadherin and Fibronectin (Figure 9H).

## 3. Discussion

GBM is the most common and lethal primary brain tumor in adults. Despite the neurosurgical intervention following standard radiation and TMZ, GBM easily recurs and results in dismal outcomes. These cells resist both radiation and TMZ. Due to the high cellular heterogeneity of GBM, an effective therapeutic strategy has to target both cancer stem cells and malignant-resistant cells [29]. In our experimental resistant cell model, the mesenchymal-like and cancer stem properties were enhanced in therapeutic-resistant GBM cells, which was attributed to activated TGF-β signaling and facilitation of the activity of ALDHs. TGF-β signaling and ALDHs are highly correlated to cancer progression [18,30], and EMT promotes cell motility, invasiveness, and even resistance to cancer therapies [31,32]. However, the interplay between TGF-β signaling and ALDH remains unclear. In certain cancers, blockade of TGF-β signaling can decrease ALDH activity [33]. In parallel, TGF-β-induced EMT promotes ALDH-modulated cancer stemness [34]. In contrast, other researchers have discovered that activating TGF-β signaling reduces the ALDH-positive cancer population [35], and inhibiting TGF-β signaling by LY364947 increases the ALDH-positive cell population [36]. In our resistant GBM model, inhibiting ALDHs did not affect the cellular behavior of parental GBM cells but reduced resistance-induced cell migration. Blockade of ALDHs inhibited the formation of tumor spheres. Additionally, inhibiting TGF-β signaling by LY364947 and Galunisertib reversed therapeutic-resistance-induced mesenchymal-like phenotype and inhibited cell motility. Exogenous TGF-β did not affect the activity of ALDHs, while LY364947 and Galunisertib downregulated ALDH activity. Conversely, inhibiting ALDHs suppressed the secretion of TGF-β of radiation-resistant and radiation-TMZ-resistant GBM cells. Furthermore, inhibiting ALDHs directly reduced the expression of TRI, Smad2, and Slug to inhibit TGF-β-induced mesenchymal-like phenotype. However, our study focused on the correlation between ADLH and TGF-β signaling in comprehensive population after experimental radiation alone or radiation followed with TMZ. In addition, although DSF was developed decades prior as an anti-alcoholism drug by inhibiting pan-ALDHs, the current studies reported that the effects of DSF may occur in an ALDH-independent manner [37]. Li et al. indicated that DSF can induce tumor cell death in an ALDH-independent manner [38]. In addition, DSF is reported as an inhibitor of NF-κB to interfere TGF-β-induced epithelial–mesenchymal transition in cancers [39]. In order to clarify the intact interaction between ADLH and TGF-β signaling, the selection of ALDH-positive subgroups and the major ALDH isoform(s) consisting of ALDEFLUOR activity need to be figured out. Also, for mechanistic investigation, gene modification, such as specific ALDH isoform knockdown/overexpression and functional mutation, is necessary. Therefore, in this study, we summarized that ALDH activity may be potentially involved in the regulation of TGF-β signaling and TGF-β-induced mesenchymal-like phenotype in this experimental therapeutic-resistant GBM model.

Otherwise, based on the above findings, DSF and Galunisertib were introduced to evaluate the therapeutic efficacy of inhibiting TGF-β signaling and ALDHs. DSF is an anti-alcoholism drug. In 2017, Karamanakos’ groups treated a GBM patient with DSF in combination with the standard of care and improved progression-free and overall survival [28]. Although DSF has been suggested as an anti-tumor agent [40,41,42], and a phase II clinical trial indicated that DSF is safe and is well-tolerated, combining TMZ and DSF did not significantly improve the prognosis of recurrent GBM patients [43]. In addition, various studies have indicated that DSF may be a good sensitizer for radio- and chemotherapy [44]. A combination of DSF and copper enhances radiosensitivity via inducing cell death or interfering with DNA repair [45,46]. Also, DSF can overcome drug resistance and potentiates anti-tumor efficacy [47,48]. On the other hand, Galunisertib is an approval drug targeting TGF-β receptors. In 2015, a clinical study indicated that Galunisertib was safe for patients with glioma [49]. However, Galunisertib in combination with standard TMZ-based radiochemotherapy or alone with lomustine did not significantly improve the survival of newly diagnosed or recurrent GBM patients [50,51]. Accordingly, combining DSF and Galunisertib may enhance the anti-tumor activity by dual targeting of cancer stem cells and malignant mesenchymal-like GBM cells. We found that combining DSF and Galunisertib showed better inhibitory effects on cell proliferation, mobility, and the growth of tumor spheres. Furthermore, due to the activation of TGF-β signaling and ALDHs by therapeutic resistance, the combined treatment had better anti-tumor effects in RR and RTR GBM cells than in parental GBM cells. In vivo, sole DSF and Galunisertib did not affect the growth of RR GBM. However, combining DSF and Galunisertib led to efficient suppression of resistant GBM growth. In spite of the in vitro and in vivo results showing the anti-tumor potential combining DSF and Galunisertib by targeting both cancer stem cells and mesenchymal-like cells, however, we only examined the inhibitory effects of combining DSF and Galunisertib by using tumor sphere formation assay. The effects of combination of DSF and Galunisertib on the self-renewal ability, the expression of cancer stem cell markers (such as CD133, SOX2, and NANOG), and the ability for differentiation into multiple cell types are necessary. Various cell-based assays have been developed for evaluating cancer stemness. For instance, secondary tumor sphere formation assay can be used to evaluating the ability of self-renewal [52]. Also, sorting out subpopulation by multiple markers is one of common manner to investigate the role of cancer stem cells in both basic and clinical cancer biology [53]. Furthermore, xenotransplantation with extreme limiting dilution assay is able to evaluate tumor-propagating and tumor-initiating abilities [54]. Therefore, investigating the effects of combining DSF and Galunisertib on the cancer stem cell properties is needed for elucidating the mechanism of combination action.

## 4. Materials and Methods

### 4.1. Cell Culture

The human glioblastoma (GBM) cell line 1306MG and U87MG were maintained in Dulbecco’s Modified Eagle Medium (DMEM, Simply) with 10% fetal bovine serum (FBS, Gibco), penicillin (100 U/mL)/streptomycin (100 μg/mL) (Simply), and gentamycin (50 µg/mL) (Thermo). The cells were incubated under a humidified atmosphere of 5% CO_2_ at 37 °C.

### 4.2. Developing Therapeutic-Resistant GBM Cell Lines

GBM cells were subjected to γ-ray irradiation by the accelerator in the Department of Radiation Oncology of Kuo General Hospital in Tainan. The median effective dose of irradiation was selected to develop the consecutive irradiation-induced resistance. Therefore, 6 times of 3.5 Gy irradiation were required for 1306MG (1306MG 3.5GR6) and 4 times of 2 Gy irradiation were required for U87MG (U87MG 2GR4). The indicators involved in radiation resistance were examined in the previos study [24]. In order to develop radiation-temozolomide (TMZ) resistance, the radiation-resistant 1306MG 3.5GR6 and U87MG 2GR4 cells were treated with 0 μM, 50 μM, 100 μM, 150 μM, 200 μM, and 250 μM TMZ to determine the dose of TMZ. After TMZ treatment, the cells with the selected dose of TMZ were amplified and consecutively treated sequential concentration of TMZ until the viability was significantly higher than the viability of the first treatment. To develop radiation-TMZ-resistant GBM cell lines, 250 μM TMZ treatments for 3 times to 1306MG 3.5GR6 (1306MG R6T3) and U87MG 2GR4 (U87MG R4T3) were required.

### 4.3. Enzyme-Linked Immunosorbent Assay (ELISA)

The cells were cultured onto 6-cm dishes. GBM cells were treated with DMSO as the vehicle control, 10 ng/mL TGF-β1, 4 μM LY364947, 100 μM Galunisertib (G), Disulfiram (8 μM for 1306MG, 150 nM for U87MG) (DSF), or NCT-501 (300 nM for 1306MG, 100 nM for U87MG), or with combined treatments. The conditioned media were collected as described previously [24]. The secretions of TGF-β1 were measured using a TGF beta-1 Human/Mouse Uncoated ELISA Kit (Invitrogen, Catalog # 88-8350-88).

### 4.4. MTT Assay

4000 cells were incubated in 96-well plate for 24 h. The cultured media were replaced with media with different doses of LY364947 or Galunisertib for 24 h. The media of post-treated cells were replaced with 0.5 mg tetrazolium salt (3-(4,5-dimethylthiazol-2-yl)-2,5-diphenyltetrazolium bromide or MTT) in 1 mL regular media for incubating for 3 h at 37 ℃ with 5% CO_2_. The insoluble crystals were dissolved in DMSO. The colored solution was quantified by measuring absorbance at 575 nm using The FlexStation^®^ 3 Multi-Mode Microplate Reader (Molecular Devices, San Jose, CA, USA).

### 4.5. ALDEFLUOR Assay

Cells were incubated in 6-well plates and were subjected to single or combined treatments. The activity of ALDH was measured using an ALDEFLOUR Kit (StemCell Technologies, Vancouver, BC, Canada). The ALDH^+^ cells were determined using CytoFlex-6 colors (Beckman Coulter, Brea, CA, USA).

### 4.6. Trypan Blue Exclusion Assay for Cell Viability

The post-treated cells were suspended with trypsin. The cell suspension was mixed with trypan blue at a ratio of 1:3. The cell concentration was counted with a cytometer, and the viability was calculated using the following equation: [(cell number of treatment)/(cell number of control)] × 100%.

### 4.7. Combination Index Calculation and Determination of Combined Effects

The combination index (CI) of combining Disulfiram and Galunisertib was calculated by using free CompuSyn software (ComboSyn, Inc., Paramus, NJ, USA). According to the cell viability curves of Disulfiram and Galunisertib, the cytotoxicity of combining Disulfiram and Galunisertib of 20% inhibiting cell viability, and half-dose of above combination, the CI values were calculated. The combined effect of combining Disulfiram and Galunisertib was determined by Chou’s group reported criteria [55].

### 4.8. Wound Healing Assay

The cells were plated in 2-chamber culture inserts (ibidi). After the cells formed a monolayer, the inserts were removed to create a wound. The cells were then subjected to different treatments in serum-free media. The wound area was observed from 0 h to different timepoints. 18 h were required for 1306MG, and 24 h were required for U87MG. The area of wound was measured using ImageJ software. The migration rate was calculated using the following equation: the percentage of wound area = [(wound area at timepoint)/(wound area at 0 h)] × 100%.

### 4.9. Transwell Assay

The cell monolayers formed in the upper chambers of the Transwell inserts (CORNING). The cells were subjected to different treatments in serum-free media for 9 h, while the 10% FBS-containing media was added in the lower chamber. The cells in the lower Transwell chamber were fixed with paraformaldehyde, and the nuclei was stained with hematoxylin (Sigma, Merck, Darmstadt, Germany), as previously described [24].

### 4.10. Tumor Sphere Formation Assay

1200 cells were suspended in the media for the purpose of forming tumor spheres, as previously described [24]. Treatments were repeated ten times with DMEM/F12 containing 10 ng/mL EGF, 10 ng/mL bFGF, and B27 was supplemented twice weekly. The cells were subjected to different treatments for 21 days. The diameters of the spheres were measured using ImageJ software.

### 4.11. Western Blotting

The cells were collected in a lysis buffer [24]. The protein expression was measured using semi-quantitative western blotting. Rabbit anti-pSmad2 (Ser465/467) (Merck, Darmstadt, Germany), rabbit anti-Smad2, rabbit anti-pTR2 (Ser225) (Abcam, Cambridge, UK), rabbit anti-TGFBR1 (TR1), rabbit anti-TGFBR2 (Santa Cruz Biotechnology, Dallas, TX, USA), rabbit anti-pTR1 (Ser165) (Aviva Systems Biology, San Diego, CA, USA), rabbit anti-Slug (Cell Signaling), rabbit anti-N-cadherin (GeneTex, Inc., Irvine, CA, USA), rabbit anti-ALDH1A1 (protein tech), rabbit anti-Fibronectin, and mouse anti-β-actin (Merck, Darmstadt, Germany) were used along with horseradish peroxidase-conjugated secondary antibody (Jackson ImmunoResearch, West Grove, PA, USA). The intensities of each band were quantified using the BioSpectrum^®^ Imaging System (Analytik Jena US LLC, Upland, CA, USA).

### 4.12. Animals

NOD.CB17-Prkdcscid/NcrCrl (NOD/SCID) mice were purchased from the Laboratory Animal Center, College of Medicine, National Cheng Kung University (NCKU). Five mice were housed in a cage at a controlled temperature (22 ± 2 °C), humidity (55 ± 5%), and a 12 h light/dark cycle. The mice were given free access to water and food. All procedures were approved by the Institutional Animal Care and Use Committee of the College of Medicine, NCKU, with project approval number (#107106 and #110013).

### 4.13. Orthotopic Xenograft Animal Model and Bioluminescence Imaging

GBM cells were stable labeled with fluorescent protein (GFP) and firefly luciferase (Luc) genes through a lentiviral infection. The cells were suspended by trypsin and PBS. After 600× *g* centrifuge for 5 min at room temperature, the suspension was removed. The cell pellet was re-suspended in PBS. Before adjusting the cell concentration, the percentage of viable cells were examined by trypan blue staining. The percentage of viable cells must be higher than 90%. 5 × 10^5^ GBM cells in 2 μL PBS were inoculated orthotopically into 8 to 10-week-old male NOD/SCID mice. The cells were injected into the right side of brain, 0.5 mm anterioposterior and 2.0 mm mediolateral to the bregma, and at a depth of 3.0 mm. DMSO served as a vehicle control, and DSF (50 mg/kg BW) (D), Galunisertib (75 mg/kg BW) (G), and DSF + Galunisertib (D + G) treatments started from post-transplantation day 10 to day 55. Tumor growth was monitored with the IVIS spectrum Live Imaging System (IVIS-200, Xenogen, PerkinElmer, Inc., Waltham, MA, USA) twice weekly, as described previously [24]. The luciferase radiance was quantitated using Live Imaging Software (Xenogen, PerkinElmer, Inc., Waltham, MA, USA) and analyzed with GraphPad Prism software.

### 4.14. Statistics

All results are presented as means ± standard error of the mean (SEM). Independent in vitro experiments were conducted using an unpaired two-tailed Student’s t-test, and a two-way ANOVA was used to analyze the differences in in vivo growth of GBM at different time points during each treatment. Significance was defined as a *p*-value less than 0.05. Microsoft Excel 2013 and GraphPad Prism6 software (GraphPad Software, San Diego, CA, USA) were used for the statistical calculation and analyses.

## 5. Conclusions

**Figure 10:**
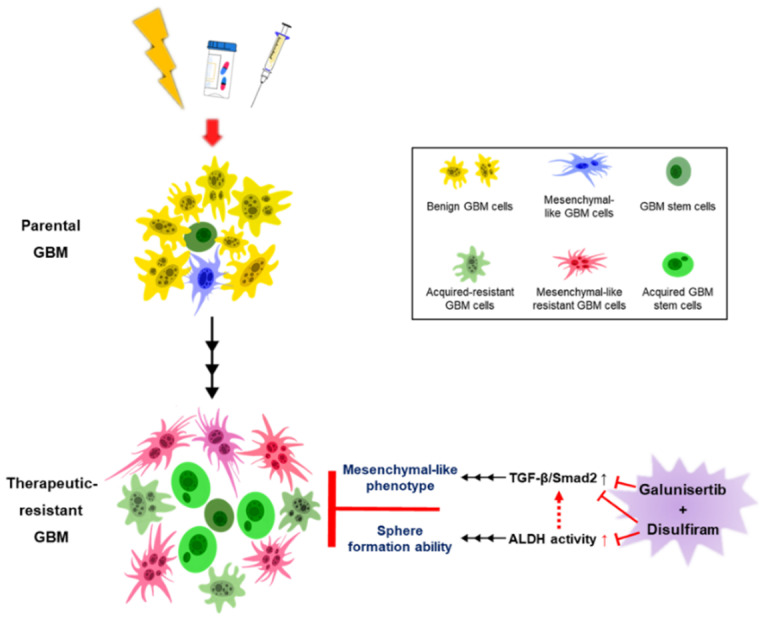
Summary of the activation of TGF-β-induced mesenchymal-like phenotype and ALDH in therapeutic resistance in GBM. Dotted red arrow means the potential interplay between ALDH activity and TGF-β/Smad2 signaling; the black solid up arrow means the development of therapeutic resistance or TGF-β/Smad2 signaling and ALDH activity-induced phenotypes; the red solid up arrow means therapeutic resistance-increased ALDH activity.

In conclusion, we developed radiation resistance and radiation-TMZ resistance in GBM cells. Dual targeting on TGF-β and ALDH showed the potential of reversing resistance-induced malignancies. Furthermore, DSF was able to desensitize GBM cells to TGF-β stimulation and was also able to sensitize therapeutic-resistant GBM cells to Galunisertib (Figure 10). Therefore, combining DSF and Galunisertib may be an effective therapeutic strategy by which to improve the prognosis of recurrent GBM patients.

## Figures and Tables

**Table 1:** The combination index (CI) of combining Disufiram and Galunisertib.

	Disufiram (DSF) (μM)	Galunisertib (G) (μM)	Cytotoxicity of DSF + G	CI	Description
1306MG	8	100	0.328	1.203	Moderate antagonism
3.5GR6	8	100	0.359	0.895	Slight synergism
R6T3	8	100	0.377	0.897	Slight synergism
U87MG	150	100	0.274	1.333	Moderate antagonism
2GR4	150	100	0.279	0.672	Synergism
R4T3	150	100	0.393	0.970	Nearly additive

The CI value was calculated by analyzing the cell viability of Disufiram, Galunisertib, and combining Disulfiram and Galunisertib with CampuSyn software. CI = 0.30–0.70, Synergism; CI = 0.70–0.85, Moderate synergism; CI = 0.85–0.90, slight synergism; CI = 0.90–1.10, Nearly additive; CI = 1.20–1.45, Moderate antagonism.

## Data Availability

The data supporting the findings and conclusion of the study are included in this manuscript. The used software is given in the Materials and Methods section.

## Supplementary Materials

The following are available online at www.mdpi.com/article/10.3390/ijms221910496/s1.

## References

[1] Stylli S.S. (2020). Novel Treatment Strategies for Glioblastoma. Cancers.

[2] Hein A.L., Ouellette M.M., Yan Y. (2014). Radiation-induced signaling pathways that promote cancer cell survival (review). Int. J. Oncol..

[3] Van Vulpen M., Kal H.B., Taphoorn M.J., El-Sharouni S.Y. (2002). Changes in blood-brain barrier permeability induced by radiotherapy: Implications for timing of chemotherapy? (Review). Oncol. Rep..

[4] Louis D.N., Perry A., Reifenberger G., von Deimling A., Figarella-Branger D., Cavenee W.K., Ohgaki H., Wiestler O.D., Kleihues P., Ellison D.W. (2016). The 2016 World Health Organization Classification of Tumors of the Central Nervous System: A summary. Acta Neuropathol..

[5] Osuka S., Van Meir E.G. (2017). Overcoming therapeutic resistance in glioblastoma: The way forward. J. Clin. Investig..

[6] Zong H., Parada L.F., Baker S.J. (2015). Cell of origin for malignant gliomas and its implication in therapeutic development. Cold Spring Harb. Perspect. Biol..

[7] Filbin M.G., Stiles C.D. (2015). Of Brains and Blood: Developmental Origins of Glioma Diversity?. Cancer Cell.

[8] Zhang Q., Feng Y., Kennedy D. (2017). Multidrug-resistant cancer cells and cancer stem cells hijack cellular systems to circumvent systemic therapies, can natural products reverse this?. Cell. Mol. Life Sci..

[9] Prieto-Vila M., Takahashi R.U., Usuba W., Kohama I., Ochiya T. (2017). Drug Resistance Driven by Cancer Stem Cells and Their Niche. Int. J. Mol. Sci..

[10] Paula A.C., Lopes C. (2017). Implications of Different Cancer Stem Cell Phenotypes in Breast Cancer. Anticancer Res..

[11] Lathia J.D., Mack S.C., Mulkearns-Hubert E.E., Valentim C.L., Rich J.N. (2015). Cancer stem cells in glioblastoma. Genes Dev..

[12] Roth P., Silginer M., Goodman S.L., Hasenbach K., Thies S., Maurer G., Schraml P., Tabatabai G., Moch H., Tritschler I. (2013). Integrin control of the transforming growth factor-beta pathway in glioblastoma. Brain.

[13] Drabsch Y., ten Dijke P. (2012). TGF-beta signalling and its role in cancer progression and metastasis. Cancer Metastasis Rev..

[14] Nana A.W., Yang P.M., Lin H.Y. (2015). Overview of Transforming Growth Factor beta Superfamily Involvement in Glioblastoma Initiation and Progression. Asian Pac. J. Cancer Prev..

[15] Bruna A., Darken R.S., Rojo F., Ocana A., Penuelas S., Arias A., Paris R., Tortosa A., Mora J., Baselga J. (2007). High TGFbeta-Smad activity confers poor prognosis in glioma patients and promotes cell proliferation depending on the methylation of the PDGF-B gene. Cancer Cell.

[16] Zhang M., Kleber S., Rohrich M., Timke C., Han N., Tuettenberg J., Martin-Villalba A., Debus J., Peschke P., Wirkner U. (2011). Blockade of TGF-beta signaling by the TGFbetaR-I kinase inhibitor LY2109761 enhances radiation response and prolongs survival in glioblastoma. Cancer Res..

[17] Bhat K.P.L., Balasubramaniyan V., Vaillant B., Ezhilarasan R., Hummelink K., Hollingsworth F., Wani K., Heathcock L., James J.D., Goodman L.D. (2013). Mesenchymal differentiation mediated by NF-κB promotes radiation resistance in glioblastoma. Cancer Cell.

[18] Martinez-Cruzado L., Tornin J., Santos L., Rodriguez A., Garcia-Castro J., Moris F., Rodriguez R. (2016). Aldh1 Expression and Activity Increase During Tumor Evolution in Sarcoma Cancer Stem Cell Populations. Sci. Rep..

[19] Li Y., Chen T., Zhu J., Zhang H., Jiang H., Sun H. (2018). High ALDH activity defines ovarian cancer stem-like cells with enhanced invasiveness and EMT progress which are responsible for tumor invasion. Biochem. Biophys. Res. Commun..

[20] Gu S., Nguyen B.N., Rao S., Li S., Shetty K., Rashid A., Shukla V., Deng C.X., Mishra L., Mishra B. (2017). Alcohol, stem cells and cancer. Genes Cancer.

[21] Rodriguez-Torres M., Allan A.L. (2016). Aldehyde dehydrogenase as a marker and functional mediator of metastasis in solid tumors. Clin. Exp. Metastasis.

[22] Januchowski R., Wojtowicz K., Zabel M. (2013). The role of aldehyde dehydrogenase (ALDH) in cancer drug resistance. Biomed. Pharm..

[23] Croker A.K., Allan A.L. (2012). Inhibition of aldehyde dehydrogenase (ALDH) activity reduces chemotherapy and radiation resistance of stem-like ALDHhiCD44(+) human breast cancer cells. Breast Cancer Res. Treat..

[24] Liu C.C., Wu C.L., Yeh I.C., Wu S.N., Sze C.I., Gean P.W. (2021). Cilostazol eliminates radiation-resistant glioblastoma by re-evoking big conductance calcium-activated potassium channel activity. Am. J. Cancer Res..

[25] Abbruzzese C., Matteoni S., Signore M., Cardone L., Nath K., Glickson J.D., Paggi M.G. (2017). Drug repurposing for the treatment of glioblastoma multiforme. J. Exp. Clin. Cancer Res..

[26] Koppaka V., Thompson D.C., Chen Y., Ellermann M., Nicolaou K.C., Juvonen R.O., Petersen D., Deitrich R.A., Hurley T.D., Vasiliou V. (2012). Aldehyde dehydrogenase inhibitors: A comprehensive review of the pharmacology, mechanism of action, substrate specificity, and clinical application. Pharmacol. Rev..

[27] Tesson M., Anselmi G., Bell C., Mairs R. (2017). Cell cycle specific radiosensitisation by the disulfiram and copper complex. Oncotarget.

[28] Karamanakos P.N., Trafalis D.T., Papachristou D.J., Panteli E.S., Papavasilopoulou M., Karatzas A., Kardamakis D., Nasioulas G., Marselos M. (2017). Evidence for the efficacy of disulfiram and copper combination in glioblastoma multiforme—A propos of a case. J. BUON.

[29] Wang Y., Li W., Patel S.S., Cong J., Zhang N., Sabbatino F., Liu X., Qi Y., Huang P., Lee H. (2014). Blocking the formation of radiation-induced breast cancer stem cells. Oncotarget.

[30] David C.J., Massague J. (2018). Contextual determinants of TGFbeta action in development, immunity and cancer. Nat. Rev. Mol. Cell Biol..

[31] Iwadate Y. (2016). Epithelial-mesenchymal transition in glioblastoma progression. Oncol. Lett..

[32] Katsuno Y., Lamouille S., Derynck R. (2013). TGF-beta signaling and epithelial-mesenchymal transition in cancer progression. Curr. Opin. Oncol..

[33] van den Hoogen C., van der Horst G., Cheung H., Buijs J.T., Pelger R.C., van der Pluijm G. (2011). The aldehyde dehydrogenase enzyme 7A1 is functionally involved in prostate cancer bone metastasis. Clin. Exp. Metastasis.

[34] Shuang Z.Y., Wu W.C., Xu J., Lin G., Liu Y.C., Lao X.M., Zheng L., Li S. (2014). Transforming growth factor-beta1-induced epithelial-mesenchymal transition generates ALDH-positive cells with stem cell properties in cholangiocarcinoma. Cancer Lett..

[35] Hoshino Y., Nishida J., Katsuno Y., Koinuma D., Aoki T., Kokudo N., Miyazono K., Ehata S. (2015). Smad4 Decreases the Population of Pancreatic Cancer-Initiating Cells through Transcriptional Repression of ALDH1A1. Am. J. Pathol..

[36] Wang Y., Jiang Y., Tian T., Hori Y., Wada N., Ikeda J., Morii E. (2013). Inhibitory effect of Nodal on the expression of aldehyde dehydrogenase 1 in endometrioid adenocarcinoma of uterus. Biochem. Biophys. Res. Commun..

[37] Schroeder J.P., Cooper D.A., Schank J.R., Lyle M.A., Gaval-Cruz M., Ogbonmwan Y.E., Pozdeyev N., Freeman K.G., Iuvone P.M., Edwards G.L. (2010). Disulfiram attenuates drug-primed reinstatement of cocaine seeking via inhibition of dopamine beta-hydroxylase. Neuropsychopharmacology.

[38] Li Y., Chen F., Chen J., Chan S., He Y., Liu W., Zhang G. (2020). Disulfiram/Copper Induces Antitumor Activity against Both Nasopharyngeal Cancer Cells and Cancer-Associated Fibroblasts through ROS/MAPK and Ferroptosis Pathways. Cancers.

[39] Han D., Wu G., Chang C., Zhu F., Xiao Y., Li Q., Zhang T., Zhang L. (2015). Disulfiram inhibits TGF-β-induced epithelial-mesenchymal transition and stem-like features in breast cancer via ERK/NF-κB/Snail pathway. Oncotarget.

[40] Lu C., Li X., Ren Y., Zhang X. (2021). Disulfiram: A novel repurposed drug for cancer therapy. Cancer Chemother. Pharm..

[41] Zhang J., Pu K., Bai S., Peng Y., Li F., Ji R., Guo Q., Sun W., Wang Y. (2020). The anti-alcohol dependency drug disulfiram inhibits the viability and progression of gastric cancer cells by regulating the Wnt and NF-kappaB pathways. J. Int. Med. Res..

[42] Xu Y., Zhou Q., Feng X., Dai Y., Jiang Y., Jiang W., Liu X., Xing X., Wang Y., Ni Y. (2020). Disulfiram/copper markedly induced myeloma cell apoptosis through activation of JNK and intrinsic and extrinsic apoptosis pathways. Biomed. Pharm..

[43] Huang J., Chaudhary R., Cohen A.L., Fink K., Goldlust S., Boockvar J., Chinnaiyan P., Wan L., Marcus S., Campian J.L. (2019). A multicenter phase II study of temozolomide plus disulfiram and copper for recurrent temozolomide-resistant glioblastoma. J. Neuro-Oncol..

[44] Wang K., Michelakos T., Wang B., Shang Z., DeLeo A.B., Duan Z., Hornicek F.J., Schwab J.H., Wang X. (2021). Targeting cancer stem cells by disulfiram and copper sensitizes radioresistant chondrosarcoma to radiation. Cancer Lett..

[45] Wang R., Shen J., Yan H., Gao X., Dong T., Wang P., Zhou J. (2020). The Evolving Role of Disulfiram in Radiobiology and the Treatment of Breast Cancer. OncoTargets Ther..

[46] Koh H.K., Seo S.Y., Kim J.H., Kim H.J., Chie E.K., Kim S.K., Kim I.H. (2019). Disulfiram, a Re-positioned Aldehyde Dehydrogenase Inhibitor, Enhances Radiosensitivity of Human Glioblastoma Cells In Vitro. Cancer Res. Treat..

[47] Rolle F., Bincoletto V., Gazzano E., Rolando B., Lollo G., Stella B., Riganti C., Arpicco S. (2020). Coencapsulation of disulfiram and doxorubicin in liposomes strongly reverses multidrug resistance in breast cancer cells. Int. J. Pharm..

[48] Jangra A., Choi S.A., Yang J., Koh E.J., Phi J.H., Lee J.Y., Wang K.C., Kim S.K. (2020). Disulfiram potentiates the anticancer effect of cisplatin in atypical teratoid/rhabdoid tumors (AT/RT). Cancer Lett..

[49] Rodon J., Carducci M.A., Sepulveda-Sanchez J.M., Azaro A., Calvo E., Seoane J., Brana I., Sicart E., Gueorguieva I., Cleverly A.L. (2015). First-in-human dose study of the novel transforming growth factor-beta receptor I kinase inhibitor LY2157299 monohydrate in patients with advanced cancer and glioma. Clin. Cancer Res. Off. J. Am. Assoc. Cancer Res..

[50] Wick A., Desjardins A., Suarez C., Forsyth P., Gueorguieva I., Burkholder T., Cleverly A.L., Estrem S.T., Wang S., Lahn M.M. (2020). Phase 1b/2a study of galunisertib, a small molecule inhibitor of transforming growth factor-beta receptor I, in combination with standard temozolomide-based radiochemotherapy in patients with newly diagnosed malignant glioma. Invesig. New Drugs.

[51] Brandes A.A., Carpentier A.F., Kesari S., Sepulveda-Sanchez J.M., Wheeler H.R., Chinot O., Cher L., Steinbach J.P., Capper D., Specenier P. (2016). A Phase II randomized study of galunisertib monotherapy or galunisertib plus lomustine compared with lomustine monotherapy in patients with recurrent glioblastoma. Neuro Oncol..

[52] Fujii H., Honoki K., Tsujiuchi T., Kido A., Yoshitani K., Takakura Y. (2009). Sphere-forming stem-like cell populations with drug resistance in human sarcoma cell lines. Int. J. Oncol..

[53] Franco S.S., Szczesna K., Iliou M.S., Al-Qahtani M., Mobasheri A., Kobolák J., Dinnyés A. (2016). In vitro models of cancer stem cells and clinical applications. BMC Cancer.

[54] Gedye C., Sirskyj D., Lobo N.C., Meens J., Hyatt E., Robinette M., Fleshner N., Hamilton R.J., Kulkarni G., Zlotta A. (2016). Cancer stem cells are underestimated by standard experimental methods in clear cell renal cell carcinoma. Sci. Rep..

[55] Chou T.C. (2008). Preclinical versus clinical drug combination studies. Leuk. Lymphoma.

