# Supplementary material for: Disulfiram Sensitizes a Therapeutic-Resistant Glioblastoma to the TGF-β Receptor Inhibitor
Source: Int J Mol Sci. 2021 Sep 28;22(19):10496. doi: 10.3390/ijms221910496 (PMC8508702; doi:10.3390/ijms221910496)
Supplement: Supplementary file 1 [file ijms-22-10496-s001.zip › ijms-1367210-supplementary.pdf]

International Journal of
Molecular Sciences

Article

Disulfiram Sensitizes a Therapeutic-Resistant Glioblastoma to the TGF-β Receptor Inhibitor

Chan-Chuan Liu 1, Cheng-Lin Wu 2,3, Meng-Xuan Lin 4, Chun-I Sze 1,3,4,* and Po-Wu Gean 1,5,6,*

1 Institute of Basic Medical Sciences, College of Medicine, National Cheng-Kung University, Tainan 701, Taiwan; sln4421@hotmail.com
2 Institute of Clinical Medicine, College of Medicine, National Cheng Kung University Hospital, National Cheng-Kung University, Tainan 701, Taiwan; wujl.towalkwithwings@gmail.com
3 Department of Pathology, College of Medicine, National Cheng Kung University Hospital, National Cheng-Kung University, Tainan 701, Taiwan
4 Department of Cell Biology and Anatomy, College of Medicine, National Cheng-Kung University, Tainan 701, Taiwan; amber002091@hotmail.com.tw
5 Department of Pharmacology, College of Medicine, National Cheng-Kung University, Tainan 701, Taiwan
6 Department of Biotechnology and Bioindustry Sciences, National Cheng-Kung University, Tainan 701, Taiwan
* Correspondence: szec@mail.ncku.edu.tw (C. I.S.); powu@mail.ncku.edu.tw (P. W.G.)

Supplementary Materials:

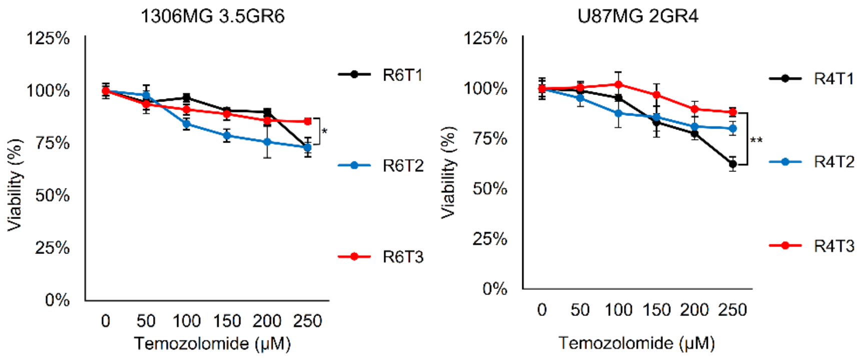

Figure S1. Developing a radiation-temozolomide-resistant glioblastoma cell model. A Trypan blue exclusion assay was performed to determine cell viability of consecutive treatment of temozolomide (TMZ). 0.1% DMSO was used as the solvent control (labeled with 0 μM). T1, T2, T3 performed as the times of TMZ treatments. The data are represented as mean ± SEM; * refers to a comparison with R6T3 or R4T3; *, $p < 0.05$; **, $p < 0.01$.

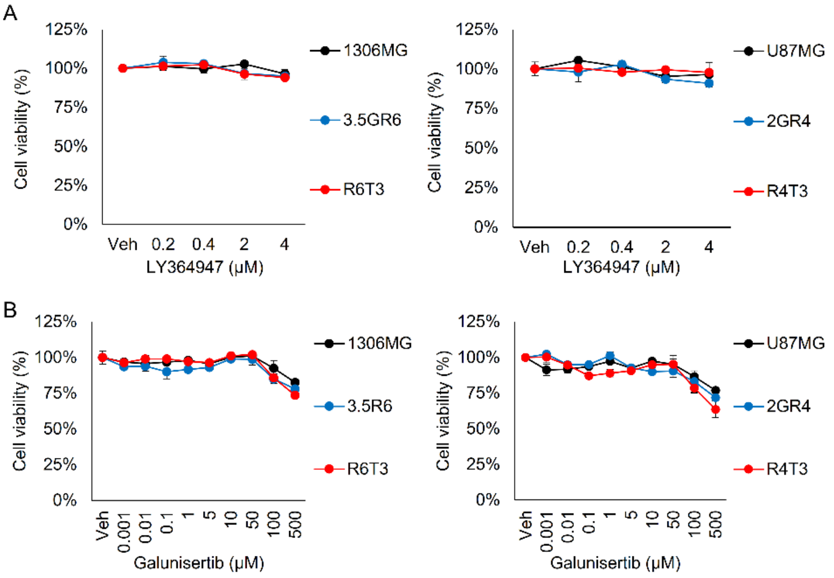

Figure S2. The cytotoxicity of LY364947 and Galunisertib. A MTT assay was performed to determine cytotoxicity of LY364947 and Galunisertib. 0.1% DMSO was used as the solvent control (labeled with Veh). (A) The cell viability curve of treating LY364947 for 24 hours; N=3 for each cell lines; (B) The cell viability curve of treating Galunisertib for 24 hours; N=3 for each cell lines; mean ± SEM.

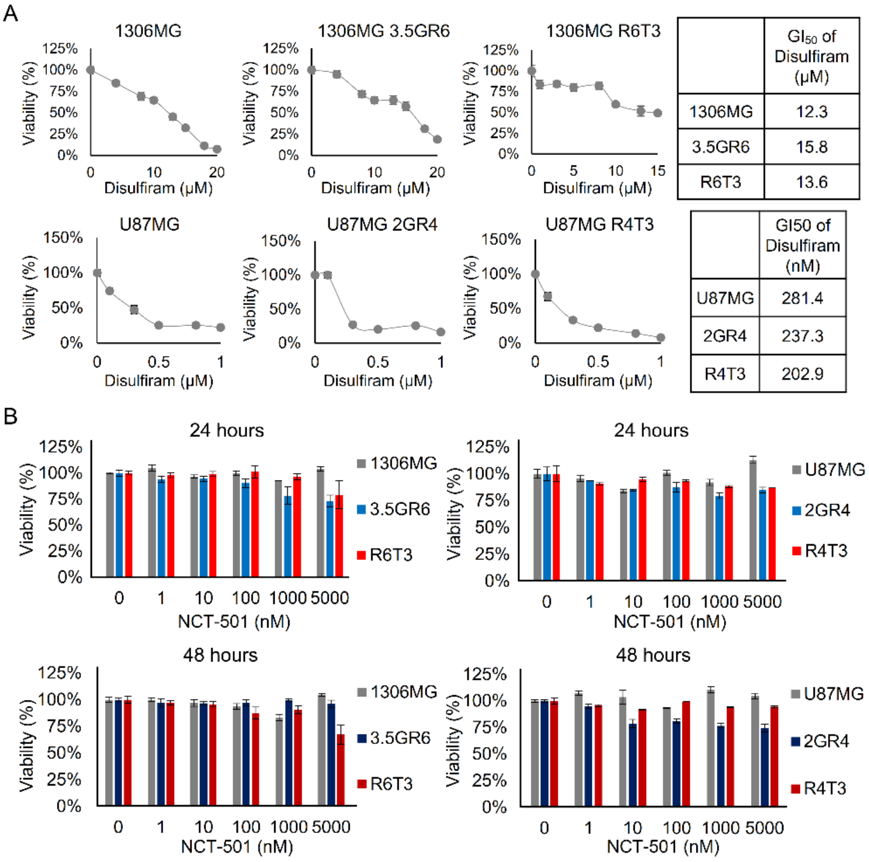

	GI_{50} of Disulfiram (μM)
1306MG	12.3
3.5GR6	15.8
R6T3	13.6

	GI50 of Disulfiram (nM)
U87MG	281.4
2GR4	237.3
R4T3	202.9

Figure S3. Cell response to pan-ALDH and ALDH1A1 inhibition by Disulfiram and NCT-501. A Trypan blue exclusion assay was performed to determine cell viability. 0.1% DMSO was used as solvent control (labelled as 0). (A) Line graphs and tables showing the effect of Disulfiram on cell viability; N=3 for each cell lines; (B) histograms showing the effect of NCT-501 on cell viability; N=4 for each cell lines; mean ± SEM.

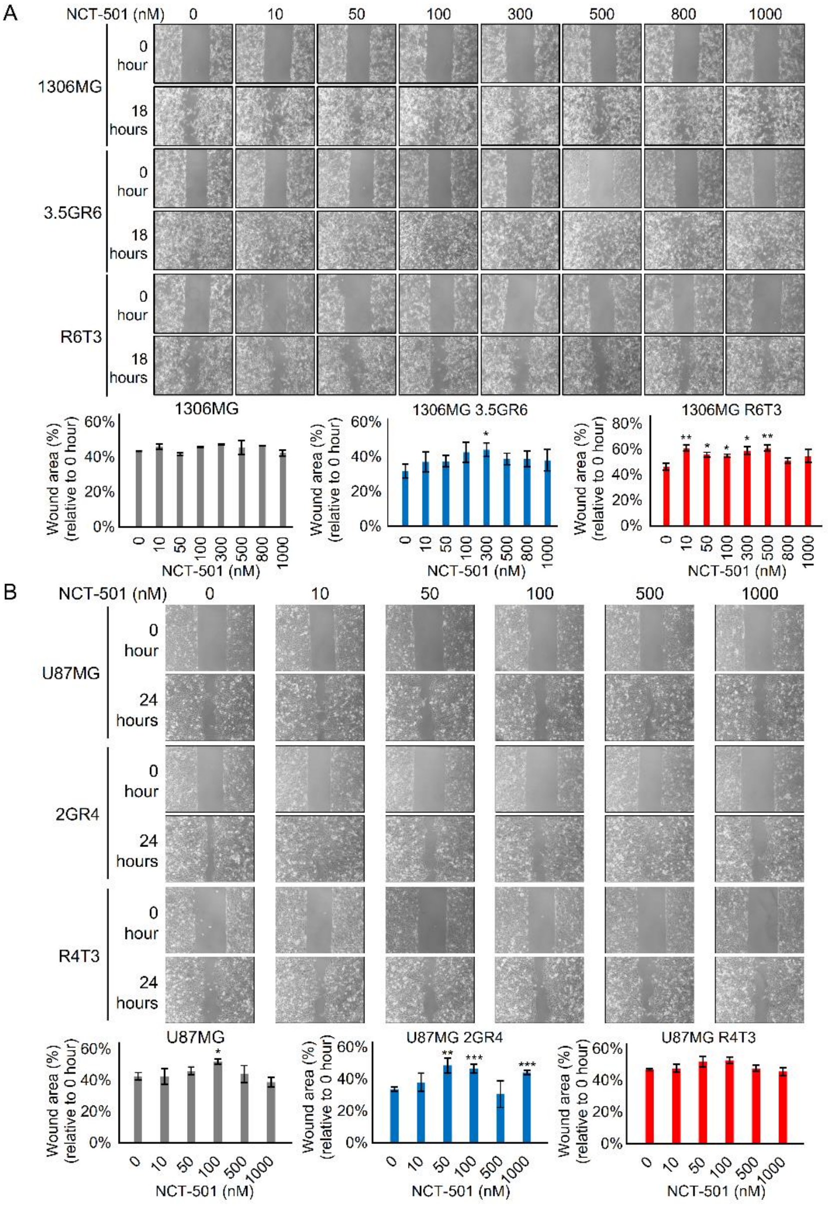

Figure S3. NCT-501 inhibited cell migration. The effect of NCT-501 on 2D migration ability was evaluated using a wound healing assay. 0.1% DMSO was used as solvent control (labelled as 0). (A) Graphs and the histogram indicating the 2D migration of 1306MG, radiation-resistant 1306MG 3.5GR6, and radiation-TMZ-resistant 1306MG R6T3 under DSF treatment; N=3; (B) graphs showing the 2D migration of parental and therapeutic-resistant U87MG; N=3; mean ± SEM; * refers to a comparison with the control; *, $p < 0.05$; **, $p < 0.01$; ***, $p < 0.001$.

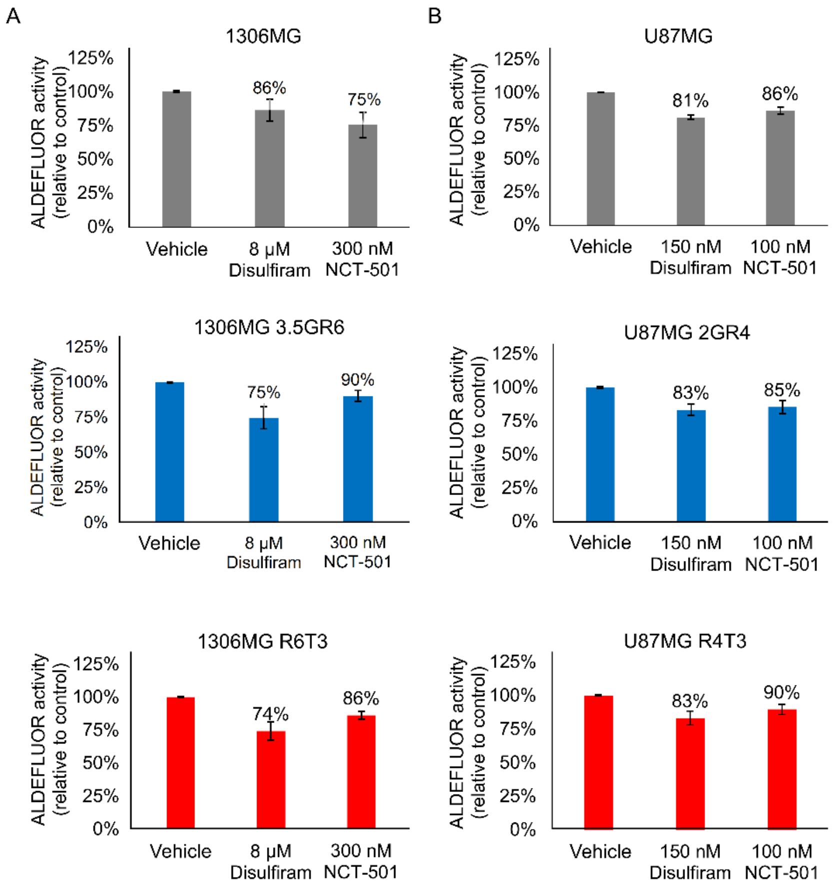

Figure S5. The inhibitory efficacy of DSF and NCT-501 on the activity of ALDH. An ALDEFLUOR assay was used to evaluate the inhibitory efficacy of selected doses of DSF and NCT-501. 0.1% DMSO was used as vehicle control. (A) Histogram showing the ALDH activity of parental 1306MG, radiation-resistant 1306MG 3.5GR6, and radiation-TMZ-resistant 1306MG R6T3; N=4; (B) histogram showing the ALDH activity of parental U87MG, radiation-resistant U87MG 2GR4, and radiation-TMZ-resistant U87MG R4T3; N=3 for U87MG; N=4 for 2GR4; N=5 for R4T3; mean ± SEM.
